# Health-related quality of life and related factors among esophageal cancer survivors after esophagectomy in the 6-month postoperative period: A multicenter cross-sectional study in north China

**DOI:** 10.1016/j.apjon.2025.100655

**Published:** 2025-01-21

**Authors:** Yingtao Meng, Ruitong Gao, Hailing Yang, Fang Zhang, Meimei Shang, Yuping Liu, Lingjuan Li, Lu Chen, Xia Zhong, Hongmei Lu

**Affiliations:** a Nursing Department, Shandong Cancer Hospital and Institute, Shandong First Medical University and Shandong Academy of Medical Sciences, Jinan, China; b The Nethersole School of Nursing, Faculty of Medicine, The Chinese University of Hong Kong, Hong Kong, China; c Qilu Hospital of Shandong University, Jinan, China; d Esophageal Surgical Department, Shandong Cancer Hospital and Institute, Shandong First Medical University and Shandong Academy of Medical Sciences, Jinan, China; e Nursing Department, The Fourth Hospital of Hebei Medical University, Shijiazhuang, China; f Nursing Department, Henan Cancer Hospital, Zhengzhou, China

**Keywords:** Esophageal cancer, HRQOL, Unmet needs, Anxiety, Depression

## Abstract

**Objective:**

Esophagectomy is a primary curable treatment and a highly challenging procedure for esophageal cancer (EC) survivors. EC survivors experience various unmet needs. This study is aimed to assess unmet needs, health-related quality of life (HRQOL), and psychological distress of postoperative EC survivors.

**Methods:**

A multicenter cross-sectional study was conducted between December 2023 and March 2024 across 28 hospitals in northern China. The European Organization for Research and Treatment of Cancer Quality of Life Questionnaire, Hospital Anxiety and Depression Scale, and Supportive Care Need Survey - Short Form 34 were utilized to assess the HRQOL, anxiety, depression, and unmet needs.

**Results:**

A total of 357 postoperative EC survivors were recruited, with a mean age of 63.42 years. Approximately 14.6% exhibited borderline anxiety, and 17.9% showed borderline depression. Unmet needs were highest in health information and patient care domains. HRQOL was lower in global health, social, and physical functions post-surgery. Fatigue, appetite loss, insomnia, and financial difficulties were common. Dysphagia, dry mouth, reflux, and choking negatively impacted HRQOL. Multivariable regression analysis indicated that anxiety and depression levels were higher, and HRQOL was lower in those one week to six months post-surgery compared to one-week post-surgery.

**Conclusions:**

EC survivors experience significant psychological distress and reduced HRQOL up to six months post-surgery. Dysphagia and unmet needs are prevalent. Compared to immediate post-surgery, EC survivors experienced higher levels of anxiety and depression, and lower level of HRQOL in six months. Future research should focus on developing individualized care strategies to provide optimal support.

## Introduction

Esophageal cancer (EC) ranks the seventh leading cause of cancer death globally, with an estimated 445,000 deaths in 2022.^1^ Over 50% of global EC cases occur in China.^2^ The incidence and mortality rates varied across different regions in China, with relatively higher rates in north–central regions.^2^, ^3^, ^4^ Surgery is the primary curative treatment for EC, often combined with radiotherapy and chemotherapy. Esophagectomy for cancer typically includes removing the main part of the esophagus for tumor resection and reconstructing the digestive tract using a gastric tube, which is pulled up into the chest or the neck and anastomosed to the remaining proximal esophagus.^5^

The average hospital stay following esophagectomy in China is 14.31 days.^6^ However, the duration is typically shorter in tertiary Grade A hospitals in China due to the implementation of the enhanced recovery after surgery and improved utilization of health resources.^7^ This aligns with a national investigation in Netherland, which reported a median length of hospital stay of 9 days (ranging from 6.5 to 12.5 days) after uncomplicated esophagectomy, with no association between length of hospital stay and readmission rates.^8^ A shorter hospital stay means that patients may not return to normal physical function and activities of daily living before being discharged from the hospital.^9^ Patients may encounter inconvenience and anxiety due to the discomfort from the surgery, treatments and altered physical function.^9^^,^^10^ Additionally, the lack of care experience may also cause anxiety during the transition from the hospital to home for patients, decreasing their quality of life.^11^

Previous studies showed that the health-related quality of life levels after esophagectomy did not return to baseline even at 6 months, and there were time-associated changes in health-related quality of life in the early postoperative phase of esophagectomy.^12^^,^^13^ Patients experienced severe deterioration in their physical, role, and social functions and suffered from various significant problems, including dyspnea, diarrhea, dry mouth, nausea and vomiting, and taste problems.^13^ Understanding the quality of life of post-esophagectomy patients in China is essential to support their recovery. Measuring quality of life not only provides data on survival,^14^ but also offers additional information that helps health professionals and patients weigh the pros and cons of different treatment strategies and make informed treatment choices.^15^ Measuring quality of life at different points in time can also help doctors, patients and families develop appropriate and targeted coping strategies. Especially at this critical time point before discharge, studies have shown that after esophagectomy, patients have not yet made plans for the future and expect to receive truthful information and better health system services, but patients have unmet needs related to their quality of life after surgery.^7^^,^^9^

The aim of this study was to investigate the level of quality of life in patients with EC in northern Chinese cities, comparing their quality of life one week after surgery, that is, before discharge, to the quality of life from one week to six months after surgery. This study also aimed to assess unmet needs and psychological distress, providing more evidence for interventions targeting patients discharged from hospitals after esophagectomy.

## Methods

### Study design and settings

This is a multi-center cross-sectional study conducted through an online survey. From December 2023 to March 2024, participants were recruited from 28 hospitals from 25 cities across 8 provinces in northern mainland China, including Shandong, Shaanxi, Shanxi, Tianjin, Beijing, Henan, Hebei, and Heilongjiang. The questionnaire link or QR code was distributed digitally via the WeChat mobile app.

### Participants

The inclusion criteria were: (1) aged 18 years or above, (2) diagnosed as EC, (3) had undergone esophagectomy in the past six months, (4) able to read and communicate in Chinese, and (5) could provide informed consent to participate in this study. Exclusion criteria were: (1) diagnosed with impairments such as dementia or mental health issues such as psychotic disorders that would affect their ability to participate in the study, (2) scheduled for transfer to palliative care as determined by their physicians. Participants who completed less than 50% of the questionnaire items were excluded from the analysis.

### Sample size

Based on the formula for determining the sample size for a multivariate analysis 50 ​+ ​8∗n,^16^ where n refers to the number of independent variables in the final model, the study included 23 independent variables. Considering a 20% incomplete rate, a sample size of 293 participants was needed.

### Study instruments

#### Demographics and clinical characteristics

This study's demographics and clinical characteristics were compiled based on previous research.^17^ In this study, participants' demographic characteristics, including age, sex, educational level, marital status, employment, insurance, place of residence, income, caregiver, smoking, alcohol, and time for physical activity were collected. Clinical characteristics included days after surgery, treatment before surgery, treatment after surgery, and comorbidity.

#### Unmet needs

Unmet needs were assessed by the 34-item Supportive Care Needs Survey (SCNS-SF34), a short-form scale revised from SCNS-SF59 by Australian scholar Boyes in 2009.^18^ It was translated into Chinese and validated by Au in 2011.^19^ The Chinese version has good reliability and validity, with Cronbach's α for each dimension greater than 0.75. The SCNS-SF34 consists of 34 items in 5 dimensions: health system and information needs (11 items), care and support needs (5 items), psychological needs (10 items), physical and daily life needs (5 items), and sexual needs (3 items), mainly measuring the needs of patients in the past month. The scoring system uses the Likert 5-point scale, with 1 indicating no need (irrelevant), 2 indicating no need (met), 3 indicating low need, 4 indicating moderate need, and 5 indicating high need. The Likert score of each dimension should be transformed into a standardized score of 0–100, with higher scores indicating higher demand.^18^ Each dimension of unmet demand can be divided into two groups: no/low demand (including not applicable/satisfied/low need) and moderate/high demand.^20^ The SCNS-SF34 has been validated in Chinese patients with EC.^7^

#### Anxiety and depression

Anxiety and depression were evaluated by the Hospital Anxiety and Depression Scale (HADS), a self-reported questionnaire widely applied in hospital and clinic settings.^21^ It consists of seven items each on anxiety and depression subscales, scored on a four-point Likert-type scale ranging from 0 (no problem) to 3 (high level of problems). The total score of each subscale ranges from 0 to 21. A total score of seven or less indicates a non-anxiety/depression case; 8 to 10 is a borderline case of anxiety/depression, and 11 or above means anxiety/depression.^22^ The Chinese version of HADS is reliable and valid, with Cronbach's α coefficient of 0.855 for the anxiety scale and 0.879 for the depression scale.^23^

#### Health-related quality of life

Health-related quality of life was evaluated by the Quality of Life-Core 30 questionnaire (QLQ-C30) and the supplemental and disease-specific Quality of Life esophageal module 18 questionnaire (QLQ-ES18), both developed by the European Organization for Research and Treatment of Cancer (EORTC) and widely used globally.

EORTC QLQ-C30 (V3.0, Chinese version) is a 30-item quality-of-life measure for cancer patients.^24^ It consists of five functional domains (physical, role, cognitive, emotional, and social), three symptom areas (fatigue, pain, nausea and vomiting), one general health area, and six single items (insomnia, appetite loss, dyspnea, diarrhea, constipation, and financial difficulties). Items are scored on a four-point Likert-type scale ranging from 1 (not at all) to 4 (very much), except for the functional areas, which need reverse scoring, and the global health scale ranging from 1 (very bad) to 7 (very good). Higher scores in the functional and general health status domains indicate better functional status and quality of life, whereas higher scores in symptoms and other problem domains indicate worse quality of life. Cronbach's α for each dimension is greater than 0.7.^24^

QLQ-ES18, developed as a subscale of QLQ-C30, is used in combination with it to evaluate the QoL of patients with EC. There are 18 items across 10 areas: pain, eating, dysphagia, reflux, saliva, swallowing obstruction, dry mouth, loss of appetite, cough, speech. Items are scored on a four-point Likert-type scale ranging from 1 (not at all) to 4 (very much), except for the dysphagia areas that need reverse scoring. Higher scores (except for dysphagia) indicate worse quality of life. The Chinese version scale is reliable, with Cronbach's α coefficient of each dimension ranging from 0.689 to 0.822.^25^ The combined use of QLQ-C30 and QLQ-ES18 (including the Chinese version) has been applied in studies of quality of life in patients with EC, demonstrating good reliability and validity.^25^

### Data collection procedures

After giving their informed consent, the participants in each hospital scanned the QR code, which contained the questionnaire link, through the WeChat mobile app to access and complete the questionnaires. The research assistants were available to answer any questions about the survey items. If the participants encountered difficulties using WeChat or reading the questions, the research assistants assisted them in scanning the QR code and reading out the questions and options without any inducement. Once they had completed the questionnaire, the research assistants verified the submission status to ensure submission was successful.

### Data analysis

Sociodemographic and clinical characteristics, along with scores for unmet need, anxiety and depression, and health-related quality of life were summarized using descriptive statistics. Continuous variables were expressed as means with standard deviation (SD), when they were normally distributed, and as medians (inter-quartile ranges) if they were not, while categorical variables were shown as frequencies and percentages. To examine differences in domain scores for unmet need, anxiety and depression, and health-related quality of life between patients with postoperative times of less than one week and those with one week to six months, univariable and multivariable linear regression analyses were conducted. Multivariate analysis was performed only if the univariable analysis yielded a *P*-value of less than 0.25. All sociodemographic and clinical characteristics were adjusted in the multivariable analysis. Mean scores with 95% confidence intervals (95% CI) were calculated and compared between the two groups. Statistically significant was defined as a *P*-value of less than 0.05. A difference of 10 points or more between means was deemed clinically relevant for health-related quality of life.^26^

### Ethical considerations

This study obtained ethical approval from the Survey and Behavior Research Ethics Committee of Shandong Cancer Hospital and Institute (Ref. No. 2023011066). Every participant was asked to provide informed consent.

## Results

### Participants' characteristics

**Fig. 1:**
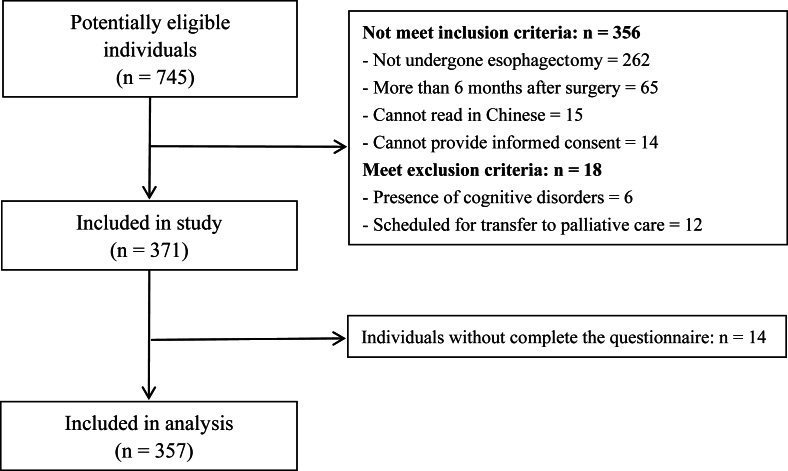
Flow diagram illustrating the process of recruitment.

We reached out to 745 EC survivors, and 371 of them agreed to participate in our study. However, 14 participants declined to finish the questionnaires (Fig. 1). Ultimately, we received 357 valid questionnaires. The results of the normality test indicate that some continuous variables were not normally distributed. The participants' characteristics were shown in Table 1. The mean age was 63.42 years (SD 9.90), with the majority being male (79.27%). Most participants had an education level of junior-middle school or below (77.03%) and were married (91.32%). Over half were working as farmers (59.95%), and a significant portion lived in rural areas (65.83%). Additionally, 66.95% had a family income of less than 3000 CNY per month. Nearly all participants had insurance and were typically cared for by their spouses or children. Nearly half of the participants engaged in physical activity for more than 30 minutes a day. A substantial percentage were current or former smokers (62.19%) and drinkers (81.23%). Regarding treatment, 38.1% were in the postoperative period of less than one week, while 38.93% and 41.46% had received chemotherapy and/or radiotherapy before and after the surgery, respectively. Furthermore, over 30% of participants had hypertension.

**Table 1:** Sociodemographic characteristics and clinical characteristics of the participants (*N* ​= ​357).

Variable	*n*	%
**Age (**Mean ​± ​SD, **years)**	63.42 ​± ​9.90	
**Sex**		
Male	283	79.27
Female	74	20.73
**Education level**		
Junior–middle school or below	275	77.03
High school	53	14.85
University and above	29	8.12
**Marital status**		
Single	26	7.28
Married	326	91.32
Widowed/divorced	5	1.40
**Employment**		
Farmer	214	59.95
Not employed/be on sick leave	28	7.84
Employed	33	9.24
Retirement	69	19.33
Others	13	3.64
**Insurance**		
New rural cooperative medical system	259	72.55
Municipal medical insurance	18	5.04
Provincial medical insurance	53	14.85
Commercial insurance	1	0.28
Others	26	7.28
**Place of resident**		
Rural area	235	65.83
Urban area	122	34.17
**Family income (Per capita monthly)**^a^		
1500 CNY or below	155	43.42
1501–3000 CNY	84	23.53
3001–5000 CNY	68	19.05
5001–10,000 CNY	47	13.16
10,001 CNY or above	3	0.84
**Caregiver**		
Spouses/partners	146	40.90
Children	196	54.90
Brothers/sisters	11	3.08
Others	4	1.12
**Time for physical activity**		
> 30 ​mins a day	172	48.18
10–30 ​mins	77	21.57
Rest most time	96	26.89
Others	12	3.36
**Smoking**		
No	135	37.81
Yes	59	16.53
Given up	163	45.66
**Alcohol**		
No	67	18.77
Yes	117	32.77
Given up	173	48.46
**Postoperative time**		
Less than 1 week	136	38.09
1week – 1 month	93	26.05
1 month–3 month	70	19.61
3 month–6 month	58	16.25
**Treatment before surgery**		
Chemotherapy	123	34.45
Radiotherapy	16	4.48
None	212	59.39
Others	6	1.68
**Treatment after surgery**		
None	194	54.34
Chemotherapy	85	23.81
Radiotherapy	19	5.32
Chemotherapy ​+ ​radiotherapy	44	12.33
Others	15	4.20
**Comorbidities∗**		
Hypertension	135	37.85
Cardiovascular disease	31	7.54
Chronic respiratory diseases	25	6.08
Diabetes	36	8.76
Others	184	44.77

*: This is a multiple-choices item, with total 411 participants.

a 1 CNY ​= ​0.14 USD (January 4, 2024; source: https://www.xe.com/).

### Prevalence of anxiety and depressive

In this study, 14.56% of EC survivors exhibited borderline levels of anxiety, and 17.93% exhibited borderline levels of depression. In comparison, 8.68% had anxiety and 11.48% had depression. Detailed data are reported in Table 2.

**Table 2:** Anxiety and depression of the participants (*N* ​= ​357).

Domain	Anxiety [*n* (%)]	Depression [*n* (%)]
Non (≤ 7 scores)	274 (76.75)	252 (70.59)
Borderline (8–10 scores)	52 (14.57)	64 (17.93)
Anxiety/depression (≥ 11 scores)	31 (8.68)	41 (11.48)

### Unmet needs

Table 3 shows that the mean score of unmet needs and health-related quality of life scores across various domains. The highest mean scores were reported in the subdomain health information needs (mean ​= ​37.81, SD ​= ​26.76), followed by patient care and support needs (mean ​= ​33.90, SD ​= ​26.40), psychological needs (mean ​= ​29.67, SD ​= ​24.54), physical and daily living needs (mean ​= ​25.81, SD ​= ​22.93) and sexuality needs.

**Table 3:** Scores of unmet needs and health-related quality of life (*N* ​= ​357).

Domain	Mean score (Mean ​± ​SD)	Range
**Needs**		
Health information needs	37.81 ​± ​26.76	0–100
Low needs [*n* (%)]	194 (54.34)	
High needs [*n* (%)]	163 (45.66)	
Psychological needs	29.67 ​± ​24.54	0–100
Low needs [*n* (%)]	234 (65.55)	
High needs [*n* (%)]	123 (34.45)	
Physical and daily living needs	25.81 ​± ​22.93	0–100
Low needs [*n* (%)]	274 (76.75)	
High needs [*n* (%)]	83 (23.25)	
Patient care and support	33.90 ​± ​26.40	0–100
Low needs [*n* (%)]	244 (68.35)	
High needs [*n* (%)]	113 (31.65)	
Sexuality needs	0 (0, 25)	0–100
Low needs [*n* (%)]	314 (87.96)	
High needs [*n* (%)]	43 (12.04)	
**General quality of life**		
Physical function	75.29 ​± ​23.41	0–100
Role function	78.01 ​± ​26.11	0–100
Emotional function	76.65 ​± ​21.66	0–100
Cognitive function	81.18 ​± ​21.13	0–100
Social function	73.43 ​± ​26.71	0–100
Global health	66.99 ​± ​21.89	0–100
Fatigue	33.12 ​± ​22.31	0–100
Nausea [median (inter-quartile range)]	16.67 (0, 33.33)	0–100
Pain [median (inter-quartile range)]	33.33 (0, 33.33)	0–100
Dyspnea [median (inter-quartile range)]	33.33 (0, 33.33)	0–100
Insomnia [median (inter-quartile range)]	33.33 (0, 33.33)	0–100
Appetite loss [median (inter-quartile range)]	33.33 (0, 33.33)	0–100
Constipation [median (inter-quartile range)]	16.67 (0, 33.33)	0–100
Diarrhea [median (inter-quartile range)]	0 (0, 33.33)	0–100
Financial difficulties [median (inter-quartile range)]	33.33 (0, 66.67)	0–100
**Esophageal cancer-related quality of life**		
Dysphagia	48.93 ​± ​35.95	0–100
Swallowing [median (inter-quartile range)]	0 (0, 33.33)	0–100
Choking [median (inter-quartile range)]	0 (0, 33.33)	0–100
Eating	22.97 ​± ​21.26	0–100
Dry mouth	32.03 ​± ​28.68	0–100
Loss appetite [median (inter-quartile range)]	0 (0, 33.33)	0–100
Cough [median (inter-quartile range)]	0 (0, 33.33)	0–100
Speech [median (inter-quartile range)]	0 (0, 33.33)	0–100
Reflux [median (inter-quartile range)]	16.67 (0, 33.33)	0–100
Pain [median (inter-quartile range)]	11.11 (0, 33.33)	0–100

### Health-related quality of life (HRQOL)

For the functional and general health status domains of EORTC QLQ-C30, the lowest scores were observed in the global health, followed by social function and physical function indicting lower quality of life in these areas. Among the symptom domains, the highest scores were reported for fatigue, appetite loss, and insomnia. Financial difficulties also presented a high level (Table 3).

For esophageal specific health-related quality of life, participants reported higher symptom scores, indicating lower quality of life, in the areas of dysphagia, dry mouth, eating, reflux and choking (Table 3).

### Univariable and multivariable linear regression analysis

Table 4 presents the univariable and multivariable linear regression analyses for unmet needs and health-related quality of life scores various domains. After performing univariable linear regression analysis on domain scores for unmet need, anxiety, depression, and health-related quality of life between patients with postoperative times of less than one week and those with one week to six months, a *P*-value of < 0.25 was found for the following domains: health information needs, anxiety, depression, physical function, role function, emotional function, cognitive function and social function, nausea, pain, dyspnea, appetite loss, diarrhea, financial difficulties, dysphagia, swallowing, eating, reflux.

**Table 4:** Univariable and multivariable analyses EORTC QLQ-C30 and EORTC QLQ-ES18 questionnaire (*N* ​= ​357).

Characteristics	Postoperative time		Univariate linear regression analysis Difference in means (95%) CI, *P*	Multivariate analysis Difference in means (95%) CI, *P*^a^
	< 1 week (*n* ​= ​136)	1 week–6 ​m (*n* ​= ​221)		
**Needs (Mean ± SD)**				
Health information needs	40.64 ​± ​27.01	36.08 ​± ​26.52	**−4.566 (−10.291, 1.159), 0.118**	−2.390 (−8.889, 4.110), 0.470
Psychological needs	29.96 ​± ​25.08	29.49 ​± ​24.26	−0.472 (−5.740, 4.795), 0.860	
Physical & daily living needs	27.21 ​± ​21.86	24.95 ​± ​23.57	−2.251 (−7.167, 2.665), 0.368	
Patient care and support	35.00 ​± ​26.56	33.24 ​± ​26.34	−1.765 (−7.428, 3.899), 0.540	
Sexuality needs	0 (0, 25)	0 (0, 25)	0.594 (−4.214, 5.401), 0.808	
**Anxiety**	4.15 ​± ​3.73	5.23 ​± ​4.19	1.076 (0.214, 1.938), 0.015	1.014 (0.105, 1.923), 0.029
**Depression**	4.45 ​± ​3.84	5.68 ​± ​4.42	1.235 (0.333, 2.137), 0.007	1.060 (0.149, 1.972), 0.023
**General quality of life**				
**Functioning**				
Physical function	78.58 ​± ​23.33	73.27 ​± ​23.29	−5.305 (−10.300, −0.311), 0.037	−4.401 (−9.646, 0.845), 0.100
Role function	80.51 ​± ​26.88	76.47 ​± ​25.56	−4.044 (−9.632, 1.544), 0.156	−3.693 (−9.747, 2.361), 0.231
Emotional function	80.39 ​± ​19.13	74.36 ​± ​22.82	−6.033 (−10.639, −1.427), 0.010	−5.664 (−10.565, −0.762), 0.024
Cognitive function	85.42 ​± ​17.97	78.58 ​± ​22.50	−6.834 (−11.313, −2.356), 0.003	−5.551 (−10.274, −0.828), 0.021
Social function	75.74 ​± ​26.90	72.02 ​± ​26.55	−3.714 (−9.433, 2.005), 0.202	−1.321 (−7.582, 4.941), 0.678
**Global health**	65.81 ​± ​23.09	67.72 ​± ​21.13	1.914 (−2.780, 6.607), 0.423	
**Symptom scores**				
Fatigue	33.17 ​± ​21.43	33.08 ​± ​22.88	−0.088 (−4.876, 4.700), 0.971	
Nausea	16.42 ​± ​20.08	23.38 ​± ​22.11	6.957 (2.379, 11.535), 0.003	3.471 (−1.136, 8.079), 0.139
Pain	25.98 ​± ​23.20	22.25 ​± ​23.87	−3.733 (−8.795, 1.329), 0.148	−1.616 (−7.104, 3.871), 0.563
Dyspnea	0 (0, 33.33)	33.33 (0, 33.33)	4.468 (−0.531, 9.467), 0.080	3.931 (−1.464, 9.326), 0.153
Insomnia	33.33 (0, 33.33)	33.33 (0, 33.33)	−0.471 (−6.243, 5.301), 0.873	
Appetite loss	33.33 (0, 33.33)	33.33 (0, 33.33)	5.053 (−0.706, 10.812), 0.085	2.397 (−3.757, 8.551), 0.444
Constipation	0 (0, 33.33)	0 (0, 33.33)	1.452 (−4.029, 6.933), 0.603	
Diarrhea	0 (0, 33.33)	0 (0, 33.33)	4.261 (−0.152, 8.674), 0.058	1.841 (−3.096, 6.777), 0.464
**Financial difficulties**	33.33 (0, 33.33)	33.33 (0, 66.67)	5.053 (−1.794, 11.900), 0.148	2.768 (−4.673, 10.209), 0.465
**Esophageal cancer-related QOL**				
Dysphagia	30.96 ​± ​35.30	59.98 ​± ​31.68	29.016 (21.920, 36.112), < 0.001	18.374 (11.155, 25.593), < 0.001
Swallowing	0 (0, 33.33)	0 (0, 33.33)	4.808 (−0.679, 10.294), 0.086	4.975 (−1.113, 11.062), 0.109
Choking	0 (0, 33.33)	33.33 (0, 33.33)	1.301 (−4.419, 7.021), 0.655	
Eating	18.75 ​± ​20.58	25.57 ​± ​21.31	6.816 (2.307, 11.324), 0.003	6.717 (1.882, 11.551), 0.007
Dry mouth	34.07 ​± ​27.95	30.77 ​± ​29.10	−3.299 (−9.445, 2.846), 0.292	
Loss appetite	0 (0, 33.33)	0 (0, 33.33)	2.922 (−2.225, 8.069), 0.265	
Cough	0 (0, 33.33)	0 (0, 33.33)	−2.922 (−8.443, 2.599), 0.299	
Speech	0 (0, 33.33)	0 (0, 33.33)	0.094 (−4.822, 5.011), 0.970	
Reflux	16.67 (0, 33.33)	16.67 (0, 33.33)	5.100 (−0.250, 10.450), 0.062	3.542 (−2.310, 9.394), 0.235
Pain	11.11 (0, 33.33)	11.11 (0, 33.33)	0.195 (−4.078, 4.467), 0.929	

EORTC, European Organization for Research and Treatment of Cancer; QLQ-C30, Quality of Life- Core 30 questionnaire; QLQ-ES18, Quality of Life esophageal module 18 questionnaire; QOL, quality of life. Variables with *P* ​< ​0.25 in univariate analyses.

a Adjusted variables: Age, sex, treatment before surgery, educational level, marital, employment, insurance, place of resident, income, smoking, alcohol, caregiver, time for physical activity.

After adjusting for all sociodemographic and clinical characteristics, the multivariable regression revealed a statistically significant difference in the means, patients in the postoperative time one week to six months showed higher levels of anxiety (difference in means 1.014, *P* ​= ​0.029) and depression (difference in means 1.060, *P* ​= ​0.023), as well as lower levels quality of life domains: emotional function (difference in means 5.664, *P* ​= ​0.024), cognitive function (difference in means 5.551, *P* ​= ​0.021), dysphagia (difference in means 18.374, *P* ​< ​0.001) and eating (difference in means 6.717, *P* ​= ​0.007). Only the difference in dysphagia reached the threshold of 10 points, making it clinically relevant.

## Discussion

This study investigated the differences in health-related quality of life, unmet needs and psychological distress among EC patients following esophagectomy, comparing data from one week post-surgery to six months post-surgery. The results indicated that the overall level of supportive care needs among patients after esophagectomy was mild to moderate, with about 30% experiencing psychological distress. Multivariable regression analysis revealed statistically significant differences in health-related quality of life domains of emotional function, cognitive function, dysphagia and eating, one week to six months post-surgery group with higher levels of anxiety and depression, lower level of health-related quality of life. Notably, only the difference in dysphagia reached the clinically relevant threshold of 10 points.

Our study showed that although unmet needs showed a downward trend, they may persist for up to 6 months. After surgery, patients exhibited various specific symptoms such as weight loss, nutritional problems,^27^ dysphagia, dry mouth, and reflux, all of which impaired their quality of life, as our results indicated. This significantly affected their daily routines, leading to functional and cognitive impairment, as well as impacting their social and emotional functions.^27^^,^^28^ This suggests that supportive care should be a long-term service during the rehabilitation of patients with EC. In this study, participants identified the health information domain as having the most unmet needs, while the patient care and support domain had a relatively higher score. These findings are consistent with previous studies,^7^^,^^29^ they could not independently solve their health problems after discharge. This suggests that the hospital staff should focus on patients' unmet needs and provide rehabilitation information about medical care and self-management during follow-up, in addition to the discharge education. This support would help discharged patients cope with new symptoms and adapt to their altered daily lives.

In this study, results demonstrated that patients experienced significantly higher levels of anxiety and depression from one week to six months postoperatively compared to just one-week post-surgery. Many patients had difficulty returning to normal lives after discharge, often experiencing dysphagia or food intolerance following digestive reconstruction surgery. These symptoms can lead to a recovery process that feels similar to pre-treatment conditions.^11^^,^^27^ Furthermore, the symptoms can significantly affect social interactions and daily activities, potentially resulting in social isolation, reduced support mechanisms, and increased depression.^11^ Approximately 66% of the patients in this study lived in rural areas, and about 68% were either employed as farmers, unemployed or on sick leave. Long-term treatment may exacerbate their financial concerns, causing increased anxiety after discharge. Additionally, they were more concerned about being a burden on their families.^30^ This suggests that the hospital staff should provide continuous psychosocial care during follow-up to help patients adapt to their altered daily lives.

Patients showed lower health-related quality of life in the domains of emotional function, cognitive function, dysphagia, and eating during the one week to six months after surgery, especially, the difference in dysphagia reached clinically relevant compared to the post-surgery within one week. Dysphagia is the most common postoperative symptom in patients with EC. Even after surgical resection of the tumor, factors such as anastomosis scarring and fibrosis can cause postoperative dysphagia. Anatomical changes in the shape and course of the gastric conduit at the distal end of the anastomosis, as well as poor peristalsis and changes in the function of the remaining stomach, may lead to dysphagia.^31^ Additionally, due to the reconstruction of the digestive tract and changes in eating habits post-surgery, transitioning from a liquid diet in the hospital to solid food at home may exacerbate the sensation of dysphagia. For instance, eating solid food like bread and steamed bread is essential for transitioning to a regular diet. These foods require esophagus expansion, which can help dilate esophageal stenosis but might also worsen the sensation of swallowing difficulty. Moreover, patients are often advised to eat smaller, more frequent meals after surgery. This increase in meal frequency may heighten their awareness of dysphagia, making eating seem more troublesome. This aligns with previous research indicating that dysphagia is a persistent long-term symptom. Studies have shown that patients who frequently experience dysphagia have higher unmet needs.^7^ Therefore, it is crucial for health care professionals to provide thorough education about dysphagia before patients are discharged. Early training and instruction on swallowing function should continue for at least six months post-discharge. This training should not only offer guidance, but also help patients manage their food intake and cope with symptoms mentally. Additionally, regular assessment of swallowing function during the follow-up visits is essential. Personalized interventions and self-management support should be provided to improve patients' quality of life. Furthermore, a study has shown that specific symptoms evolve over time following curative treatment for EC.^17^ Understanding patients' quality of life during follow-up is crucial to offering targeted intervention for symptoms as they arise.

### Limitations

This study comprehensively investigated health-related quality of life using standard general and cancer-specific health-related quality of life assessment tools among EC survivors within six months post-esophagectomy period across multiple centers in north China. However, several limitations should be considered. Firstly, the cross-sectional design limited outcome assessment to a specific point in time, preventing us from evaluating the trajectory of health-related quality of life in discharged patients and thus from identifying causal relationships. Secondly, we could not rule out the selection bias as we were unable to collect data from participants who declined to participate, who may have had varying levels of health-related quality of life and unmet needs. In addition, the health-related quality of life tool relied on patient recall of past events, making the responses susceptible to recall bias. Furthermore, our study may be affected by survivorship bias, as the participants might represent a select group of EC survivors who have experienced positive outcomes, potentially leading to an overestimation of the overall HRQOL in this population. Therefore, the findings should be interpreted with caution.

## Conclusions

This study investigated the differences in health-related quality of life, unmet needs and psychological distress among EC patients up to six months post-surgery in northern China. The overall level of supportive care needs among patients after esophagectomy was mild to moderate, with about 30% experiencing psychological distress. Compared to immediate post-surgery period (within one week), patients one week to six months post-surgery exhibited higher levels of anxiety and depression, and lower levels of health-related quality of life in domains such as emotional function, cognitive function, dysphagia and eating. The results highlight the importance of understanding how specific symptoms evolve over time after surgery treatment. Future research should focus on developing individualized care strategies to provide optimal support for these patients.

## CRediT authorship contribution statement

**Yingtao MENG** and **Ruitong GAO**: Study design, Data curation and writing manuscript. **Hailing Yang, Fang ZHANG, Yuping Liu, Lingjuan LI, Lu CHEN** and **Hongmei LU**: Data collection. **Xia ZHONG** analyzed and interpreted the data. **Meimei SHANG**: Conceptualization, Methodology, Data collection, revised the manuscript for important intellectual content. All authors read and approved the final manuscript. All authors had full access to all the data in the study, and the corresponding author had final responsibility for the decision to submit for publication. The corresponding author attests that all listed authors meet authorship criteria and that no others meeting the criteria have been omitted.

## Ethics statement

The study was approved by the Survey and Behavior Research Ethics Committee of Shandong Cancer Hospital and Institute (Ref. No. 2023011066) and was conducted in accordance with the 1964 Helsinki Declaration and its later amendments or comparable ethical standards. All participants provided written informed consent.

## Funding

This study was supported by the Shandong Province Medicine Science and Technology Development Plan Project (Grant No. 202214050797) and the Research Project on Education and Teaching Reform of Shandong First Medical University (Grant No. XM2022164). The funders had no role in considering the study design or in the collection, analysis, interpretation of data, writing of the report, or decision to submit the article for publication.

## Declaration of competing interest

The authors declare no conflict of interest.

## Data availability statement

Data are available from the corresponding author upon reasonable request.

## Declaration of generative AI and AI-assisted technologies in the writing process

No AI tools/services were used during the preparation of this work.
